# Of robots and humans: Creating user representations in practice

**DOI:** 10.1177/0306312720905116

**Published:** 2020-02-10

**Authors:** Björn Fischer, Britt Östlund, Alexander Peine

**Affiliations:** Department of Biomedical Engineering and Health Systems, Royal Institute of Technology KTH, Sweden; Copernicus Institute of Sustainable Development, Utrecht University, The Netherlands; Department of Biomedical Engineering and Health Systems, Royal Institute of Technology KTH, Sweden; Copernicus Institute of Sustainable Development, Utrecht University, The Netherlands

**Keywords:** design practice, engineering culture, robots, user image, user representation

## Abstract

In this study, we explore the constitution of user representations of robots in design practice. Using the results of ethnographic research in two robot laboratories, we show how user representations emerge in and are entangled with design activities. Our study speaks to the growing popularity of and investment in robotics, robots and other forms of artificial intelligence. Scholars in Science and Technology Studies (STS) have shown that it is often difficult for designers and engineers to develop accurate ideas about potential users of such technologies. However, the social context of robots and design settings themselves have received significantly less attention. Based on our laboratory ethnographies, we argue that the practices in which engineers are engaged are important as they can shape the kind of user images designers create. To capture these dynamics, we propose two new concepts: ‘image-evoking activities’ as well as ‘user image landscape’. Our findings provide pertinent input for researchers, designers and policy-makers, as they raise questions with regards to contemporary fears of robots replacing humans, for the effectiveness of user involvement and participatory design, and for user studies in STS. If design activities co-constitute the user images that engineers develop, a greater awareness is needed specifically of the locales in which the design of robots and other types of technologies takes place.

## Introduction

In the sociology of technology, a number of works have focused on the relationship between designers and users. The active role of users in shaping technology is seen in domestication literature (Lie and Sørensen, 1996; Silverstone and Haddon, 1996; Silverstone and Hirsch, 1992), studies of the social construction of technology (Bijker, 1987; Kline and Pinch, 1996; Pinch and Bijker, 1984) and user innovation research (Fleck, 1988; Hienerth, 2006; Rice and Rogers, 1980; von Hippel, 1976, 1986). In different shades, these streams of analysis highlight the agency of users to define, modify and change technologies as they incorporate them in their homes, attribute different meanings to them and invent or reinvent new uses given their particular context. At the same time, the important role of designers has been stressed in the semiotic tradition of STS (Akrich, 1992, 1995; Latour, 1991; Woolgar, 1991) and by feminist technology studies (Clarke, 1998; Cockburn and Ormrod, 1993; Wajcman, 1991). Those studies examine the possibility of power imbalances between designers and users. They refer to the subordinate position of users ‘implicated’ in design where designers often attempt to ‘configure’ the user and ‘inscribe’ certain user images into technology design.

Though available technologies are not necessarily completed products and though users can play an active role in adjusting them in various stages, the ideas of designers that are implemented into the technology can steer user behavior in certain directions while discouraging other behaviors (Akrich, 1992; Winner, 1980; Woolgar, 1991). Users can react in unpredictable ways, but their possibilities for action are enabled and constrained by these images embedded in the technology, as well as by other contextual factors influencing their position alongside the user-technology nexus (DeSanctis and Poole, 1994; Oudshoorn and Pinch, 2003). In the absence of complete information, designers are often in a prefiguring position as they need to develop some representation of the user whenever they develop a new technology (Akrich, 1995; Hyysalo and Johnson, 2016).

The question then arises how designers and engineers create such images of users in technology projects. STS research has a fairly rich tradition in critically interrogating the sources engineers and designers utilize for envisioning the user (see Peine and Herrmann, 2012). However, how engineers differently piece together their knowledge from a variety of available sources has largely been taken-for-granted. When we think about the emergence and design of new technologies, we realize that we know little about the processes by which user images are created in different design settings. That is, we lack empirical and theoretical understanding of how design is shaped by the practices and contexts in which technologies are developed. Our study asks: How do engineers imagine prospective technology users in design practice? How do user representations come into being? To answer these questions, we ethnographically investigated the work of engineers at two robot laboratories over a period of six months, and critically examined how user representations were created.

## Theory

We built our inquiry on concepts from user research in STS and the ‘semiotic approaches to users’ in particular (Oudshoorn and Pinch, 2008: 548). At the core of semiotic user research lies the position that designers hold certain preconceptions about users and therefore design in ways that would encourage real users to behave in accordance with these preconceptions. Hence, Woolgar (1991) has shown how usability trials of a microcomputer were organized so that users would behave ‘correctly’, that is, in accordance with what the designers expected them to do. Machines, according to Woolgar, are written in ways that make certain forms of their usage more likely than others; ‘the evolving machine effectively attempts to configure the user’ (Woolgar, 1991: 61). Akrich (1992) proposes the notion of a ‘script’ to capture how technologies may impact users’ actions and interactions. Like Woolgar, she argues that engineers ‘construct many different representations of … users, and objectify these representations in technical choices’ (Akrich, 1995: 168). These objectifications, according to her, work like a ‘film script’: They allocate specific roles and frames of action to both users and technologies (Akrich, 1992: 208). In contrast to Woolgar, Akrich is explicitly interested in understanding how user representations and objects evolve as they move between the worlds of users and designers. She emphasizes not only how designers would inscribe images of users, their ‘user representations’ (Akrich, 1995: 168), into a technology, but also how users would de-inscribe and renegotiate such scripts.

Having recognized the important role of the designers’ user representations in technology design, several STS scholars have shed light on the different sources of such user representations. Akrich (1995) distinguishes two different categories of sources she encountered during her research: *explicit* user representation techniques, that attempt to gain information directly from the user, such as market surveys, customer testing and feedback on experience, and *implicit* ones, that represent the users through articulations about the users by someone else, such as experts, other products, and engineers’ reliance on their own personal experiences (the latter referred to as ‘I-methodology’). Following Akrich’s initial categorization of user representation techniques, significant attention has been paid to the different sources that designers have at their disposal, and increasingly comprehensive overviews of various ideal-typical user representation sources have been produced (Hyysalo, 2009: 732; Peine and Herrmann, 2012: 1503; Williams et al., 2005: 112).

In theorizing the constitution of user representations, however, the everydayness of engineering practices and design settings have received less attention. Why are *these* sources mobilized in practice, and *these* representations formed? Only a few studies have addressed such questions and examined how user images are forged and molded in practice. In an investigation of how engineers designed a wrist-worn safety device for elderly users, Hyysalo (2006) displays the different professional traditions that bounded images of users and use. He analyzes how the interactions and bargaining between these traditions (or practices, as he calls them) gave rise to the representation of a technology user aged 60 or above, with gradually increasing health problems but a desire to remain active at home. In this view, user representations can be linked to practices that bring with them implicit ideas for imagining scenarios for users and use. Hyysalo’s work makes apparent how such an environment of practice-bound but implicit user images may be particularly forceful, as it is seldomly challenged or even explicitly discussed in design. However, Hyysalo’s findings also suggest that we still lack understandings of the dynamics by which user representations are created in actual design practice, highlighting that more work is needed to unravel those dynamics (Hyysalo and Johnson, 2016).

There are also a number of other noteworthy studies that have examined how user representations are created. For example, Pollock et al. (2003) explore the practices by which the user representations embedded in a resource planning software were adjusted as the technology travelled to new organizational settings. Specifically, they show how the image of a ‘generic user’ relating to a general market of standardized enterprises was translated into a new ‘specific’ user of universities, and then again re-adjusted into a ‘generic’ user referring to the global market of universities. Oudshoorn et al. (2004) show how a group of designers imagined how they themselves would use ICT technology and inferred, often unconsciously, that this would apply to every user. Bardini and Horvath (1995) historically trace how representations of personal computer users have been socially constructed, influenced by broader cultural and political discourse. And Neven (2010) shows how, for the design of one companion robot for elderly users, ageist stereotypes were more influential than was actual information obtained from test users .

These studies have investigated the emergence and shaping of user representations in a variety of design cases, and they have brought into sight the possibility that user representations can be bound to professional design practices. However, there has been a tendency to treat user representations as ontologically separate and separable from design practices. The general sentiment seems to have been that user representations can be studied individually, by identifying their different sources and contingencies, such as traditions, market settings, discourses or stereotypes. What appears to fall out of the analytical scope is the finegrained analysis of how user representations may be related not only to particular identifiable sources, or bound to professional traditions, but also *co-constituted with everyday design practices*. To shed light on this phenomenon, thorough on-site studies are necessary, with a particular concern for the practices of design and their epistemic and material qualities. In our study, we aimed to zoom in on the intricate dynamics of design activities and ask: How do designers differently knit together different types of knowledge about the users? How are images of users made in design practice? How are they entangled with and shaped by the other activities designers are involved in on a daily basis? We devised our study to address these questions and specifically illuminate how user representations are built in engineering practice and entangled with other design activities.

## Methodology

We have chosen an inductive and interpretivist research design in the tradition of grounded theory (Glaser and Strauss, 1967; Strauss and Corbin, 1990) and adopted ethnography as our main method of data collection (Hammersley and Atkinson, 2007). We selected our cases based on their theoretical relevance, that is, theoretical sampling (Glaser and Strauss, 1967). Both cases needed to present us with the opportunity to observe technology design in action (Latour, 1987), and we chose two laboratories at a major European institute of technology that were involved in the development of robots (referred to here as *the speech department* and *the robot department*). Following Akrich’s (1995) distinction between explicit and implicit user representation techniques, we chose the speech department because engineers were designing explicitly for a specific user group, older users, and we chose the robot department because the engineers were not explicitly concerned with users (it will become clear that they were so implicitly). Thereby, we were able to theorize the constitution of user representations in a way that is robust across explicit and implicit concerns for technology users.

We collected our main data by means of ethnographic fieldwork at the two laboratories for six months, where one of us (BF) conducted 19 field visits (five visits to the speech department and fourteen visits to the robot department). Each visit consisted of between two and five hours of field work. Throughout the period of observation, BF kept detailed field notes. To complement field observations, we developed an unstructured interview schedule that could be discussed with the engineers during informal conversations. For most of the time, the ethnographer remained silent and followed the engineers through the departments to obtain a sense of the type of activities the engineers were participating in, and the type of technologies they were developing. Based on this evolving understanding of what was going on in the departments, the observer occasionally developed the feeling that certain assumptions lingered unarticulated in the background. In such cases, the ethnographer asked questions to prompt the engineers to make these user images explicit. In this way, we aimed to obtain additional data that directly related to the situations we observed, while trying to avoid leading the engineers into a particular way of thinking or responding. For the same reasons, the observer also conducted two semi-structured, formal interviews with two engineers in the robot department, each of which lasted for about one hour. To gain a deeper sensitivity to the context, we complemented our field notes and interview data by reading academic articles in robotic engineering to which engineers frequently referred, browsed pertinent websites on robot software, and also had regular email exchanges with the two department heads.

We constantly compared observations and emerging ideas with existing literature (Glaser and Strauss, 1967). About two months into our fieldwork, we realized that we were not able to understand the ways user representations emerged in our ethnographic observations using the theories we had available at that time. Therefore, simultaneous with repeated visits, we began to engage with several different theories to think about our data. The theories we found most useful were practice theory (Bourdieu, 1977, 1990; Giddens, 1984; Schatzki, 1996, 2001), and activity theory in particular (Engeström, 1987, 2000; Leont’ev, 1978). Practice theory views practices at the core of social fabric and treats practices and social structures as mutually constitutive. Specifically, we took from those two theories an understanding of an ‘activity’ or practice as an object-oriented, collective endeavour that can be distinguished by its motive. In each activity, a variety of subjects purposefully act and interact to achieve one specific motive. The subjects need not necessarily be aware of that motive – rather, motives and goals often become clear while performing the activity (Lave, 1988). The overall motive of an activity is achieved through a range of individual sub-actions (Engeström, 1987; Schatzki, 2005). Due to this overarching nature of activities, each individual can be involved in several different activities at once (Reckwitz, 2002). We also adopted the idea that each activity is mediated by the use of various artifacts, both mental (such as mind and knowledge) and physical (such as human body, tools and technical objects), and influenced by the broader community characterized by certain sets of rules and a division of labour (Engeström, 1987; Knorr Cetina, 1997; Latour, 1992, 2005; Suchman, 2007). Activity theory has been applied substantially as a framework for analyzing empirical settings, such as human-computer interaction research (Kaptelinin and Nardi, 2006; Kuutti, 1995) and inter-organizational learning (Engeström, 2001). We observed that the emergence of different user representations was related to different types of such activities in which the engineers were engaged, and so we began to use these concepts to organize our observations of the engineers’ work.

In a final step, we pulled together the insights we had gained about both our observations and our emerging theoretical understanding. This effort involved selecting and molding our developed data for the theoretical perspective we wished to take while maintaining the essence of our observations. This step was necessary as ethnographic data can be of interest to many different theoretical strands, and the complexity and messiness of field notes requires them to be recast and re-written to provide some type of order with respect to their analytical purpose (Van Maanen, 1988). Hence, for the presentation of our results, we transformed and polished elements of our field notes to illustrate how each observed activity played out as perceived by the observer. For each activity, we provide one such ‘thick description’, adjusted based on the original corpus of field notes (Geertz, 1973). In an attempt to preserve the ‘voice’ of the engineers, we also present some snippets from our field notes which we left unpolished, such as overheard conversations or discussions. Through this presentation of results, we believe, we can show how the activities unfolded in front of the eyes of the observer in the laboratory, while maintaining how they were related to dialogue and talk as articulated by the engineers and evidenced in the original corpus of our field notes.

We should note that, in the robot department, we were allowed to observe how the engineers worked at their robots in their laboratory. Therefore, the corpus of our field notes in the robot department embodied descriptions of scenes and conversations that occurred during the presence of the observer. In the speech department, by contrast, we were allowed to observe the engineers’ regular meetings during which they discussed how to develop their robot technology. Hence, the field notes of our work in the speech department encompassed mostly notes of dialogue in work meetings. Despite this distinction, we find that the sets of field notes are comparable: Our descriptions in the robot department often involved dialogues, and most of our notes of dialogue in the speech department clearly related to work actions. Inasmuch as activities and practices consist in sets of both bodily and mental performances (Engeström, 2000; Reckwitz, 2002), we contend that forms of discussions, meetings, articulations and conversations also constitute elements of such activities. Indeed, we could find evidence of similar activities in both speech and robot laboratory, as we will show below.

## User representations in practice

In the robot department, the engineers were working on several prototypal robots, among them one ‘Baxter’ robot, one ‘Kuka’ robot, three ‘YuMi’ robots and one ‘PR2’ robot. These robots were commercially manufactured and crudely resembled frames of human bodies. The engineers at this department were concerned with advancing technical and practical knowledge about robotics. At the speech department, the engineers explored the interaction between social robots and humans, and specifically focused on developing software for speech recognition and hearing. For this purpose, they were working with a robot head in the shape of a human head, with realistic and agile eyes, eyebrows, mouth and nose. Software enabled this robot to animate its facial expressions and speak with different voices. During our period of observation, the engineers specifically aimed to develop the robot head for older users.

In the following, we present our results of four activities that we found played a role for the engineers’ user representations at both departments. We have given the engineers fictive names to ensure anonymity. Each section begins with an ethnographic account of the activity, followed by some elaborations and further quotes from our fieldnotes. Three activities were discovered in both departments, one only in the robot department. We will return to this in the discussion.

### Proficiency demarcation

I sit on a chair in one corner of the laboratory. An assortment of robots are distributed across the room, scattered, bulky and, for the moment, unused. Black wires connect them to computer bodies and sockets and blank monitors on work desks at each wall of the laboratory. The room is brightly lit, the windows are tightly shut as though in quarantine, and I can smell a faint scent of dry plastic and electric circuitry lingering in the air. There is a constant hum of machinery in the background, a muted sound of robots quietly processing, computers silently calculating and hardware cores cooling.

A handful of engineers move about, clear off shelves and corners stuffed with home utensils, carry household appliances to the middle of the laboratory, and continue to search for more, studiously rummaging around translucent plastic boxes brimming with children’s toys, kitchen tools and living room decor. Card games and lego bricks fly across the air as they are tossed around and dug out of cupboards and boxes. Beach novels and old Toblerone bars accumulate in the center, flung seemingly careless to the ground. Gradually, they are buried underneath layers of rolls of toilet paper, stacks of frying pans and batches of knives and forks, mounting as if a massive landfill. The floor, otherwise grey and replete with dispersed strips of charcoal duct tape, clutters with a jumble of objects that can easily furnish a three-person apartment.

Circling around the clamps of utensils, the engineers pause. ‘So we can use these shelves for robot stuff instead ….’ – ‘What was the plan with the utensils?’ – ‘My plan was to burn it, but … [Ivana] was not really fond of that idea.’ … Item by item, the heap of home appliance slowly disappears as the engineers set off to move them out of the laboratory and down the staircase.

The above account describes how the engineers slowly changed the laboratory’s interior as they moved home living pieces out of the laboratory to make place for robot equipment. The engineers actively worked to delineate their technical realm from a non-technical sphere, an activity that we would call ‘proficiency demarcation’. Earlier, we had observed numerous episodes in which the engineers discussed and argued with supervisors and colleagues about how home utensils could be eliminated and which new robot technology should be purchased. We observed sequences in which new technologies were ordered, received and unwrapped – amongst others, a mocap (motion capture) system, Kinect cameras, force torque sensors, and stronger robot hands. And, in the aftermath of our presented account, we made observations of how the engineers assorted those new technologies into the laboratory racks. There seemed to be a pervasive notion that technology development is serious business and should be regarded as separate from the homely signifiers of its use, as this piece of an informal conversation reveals:

Giulia: ‘And this plant, why is this plant here? Put that on the list!’

I asked: ‘Why do you not want it?’

Giulia: ‘This is a laboratory!’ …

[I] asked: ‘Why is here a plant in the first place?’

Fabian replied: ‘This robot room has been used to be a home living lab in the past.

They still call it living room for this reason. They also had two couches here in the middle.’

I wanted to know: ‘Why is it no more decorated like a living room?’

Fabian: ‘It simply became more robots, which we needed, and now it is more like a

real laboratory.’

I asked again: ‘So, why do you no more consider the living room?’

Fabian replied: ‘This place is about technology, not about the use.’

(excerpt from observed conversation – robot department)

Apparently, the engineers perceived a laboratory with ‘living room’ elements as somewhat less than a ‘real’ or first-rate laboratory. This place was meant to be for ‘technology’, which was seen as something very different from the ‘use’ context.

Similarly, in the speech department, the engineers vigorously distinguished between technical and non-technical expertise. For example, in one meeting, the engineers were talking about different options of how to configure the look of a robot head. The observer became curious and engaged in the conversation:

I asked: ‘Why do you think that an older person prefers a robot that looks like him?’.

Matthew and Adriana simultaneously responded: ‘That’s the thing … we don’t know it, that’s why we have to test it.’

I asked: ‘Why don’t you ask the older people themselves before starting the design what they prefer?’

Adriana: ‘Because when you ask them before, they might say they like this version, but then… when they see how it would look on the actual robot, they might think differently. So it’s better to test it with a given face, so it is closer to reality…’

Matthew added: ‘… and we have experts here to analyze it!’

…

Oscar: ‘And then, me and … [Matthew], we thought to do some kind of crowdsourcing to evaluate which face they prefer.’

Matthew: ‘… like, show them two different options and ask them, which is more trustworthy, what do they prefer more.’

(excerpt from observed conversation – speech department)

Using their own technical expertise as legitimation, the engineers preferred to maintain control over the development of the robot head. Specifically, Adriana argued that the older people would not know how the ‘actual robot’ would look, which is why it would not make sense to ask them about what kind of appearance they would prefer before showing them a finished version of the robot head. Instead, the engineers saw themselves as having such technical expertise for design and analysis, and therefore deemed it legitimate to prescribe their test users two options from which they could choose, with one option looking a ‘normal’ age and another option looking ‘older’.

Our continued observations made apparent how the activity proficiency demarcation was intricately tied in with the way the engineers imagined users: As our first excerpt shows, the engineers created labs that were free from vignettes of real-life use scenarios, because in their understanding, real or top-notch laboratories would be purely technical environments. Our second excerpt then shows how this purification activity implicitly encouraged the notion of users as outsiders to these environments, users who would be fairly incompetent and rather inept to provide the engineers with valuable ideas about how a robot face could look like.

Besides the specific imagery of incompetent users, our empirical work also revealed that proficiency demarcation additionally worked to increase the legitimacy of the user images provided by specialist ‘research groups’, ‘user experts’ or ‘therapists’. For example, in a formal face-to-face interview with one engineer from the robot department, we learned about software that the engineer developed for a robot in an elderly care facility to play hiking music according to the surroundings. Asked why he decided to include these ideas into the design of the robot, he replied:

Erik: ‘Ahm that’s … Actually, I think it was the therapists who, who had that idea, apparently, that’s kind of part of this Austrian tradition of hiking and having these hiking songs … and so they thought that that would be something that resonated with the inhabitants there …’

(excerpt from interview – robot department)

Later in the interview, Erik emphasized that the therapists’ advice was quite valuable, since therapists ‘have a lot more time’ and ‘are observing the robot in action with the people there’. Interestingly, Erik adopted the images offered by the therapists as an inspiration for his further work without questioning their accuracy. This example shows how proficiency demarcation co-constituted user images supplied by specialists: Users were located in an arena beyond the boundaries so carefully established during proficiency demarcation. To let ideas about users into the laboratory, specialists were allowed to cross these boundaries. At the same time, proficiency demarcation effectively made the presence of users in the flesh inadequate, and thereby implicitly continued to constitute user images around notions of incompetence.

In sum, in both departments, we observed how the engineers were frequently performing actions to mark off their technical field of interest from the non-technical world. This activity of proficency demarcation collaterally motivated implicit ways of imagining users. In our case, proficiency demarcation related to images of somewhat technically incompetent users, and it rendered user images that stemmed from experts legitimate.

### Expanding technological possibilities

To my left, two engineers, Tiago and Patricio, sit at a desk, seemingly concentrating and alternately gazing at three different monitors that are somehow connected to one and the same keyboard. On the monitor in front of them, white, flashing letters appear on an otherwise black screen as one of them continuously digs into the keyboard, two-handed and so heartily that the keys crackle under his curled fingers. ‘I noticed that it really needs to be much more automated than it is right now’, Tiago says. He moves the computer mouse to the right and periodically clicks with his right forefinger. A tiny white arrow on the screen responds, closes some document, opens another one, displays search engine results, readme files, github and other pages about robot operating systems. Subsequently, he moves the arrow to highlight some coded text on an online forum, returns back to the main monitor, deletes some written text from the compiler and replaces it with the highlighted code.

All this while, a PR2 robot stands still behind them, grey and white plastic surface, at a height of about my hips. It has a camera on its head, is stuck in half of a movement of turning the head, and a pen is held arms-length by its right gripper. Patricio stands up, lifts his hand and holds a black and white checked paper towards the camera. The robot rattles but remains still. A red error message appears on the screen. Tiago and Patricio look at each other and begin to discuss. ‘The goal is that the robot knows where the camera is …. Once you calibrated the camera, you can calculate the specifics of each object that it holds.’ – ‘For the april tag marker, there will be already the right frame from the company online… so I just need to recode a small piece by referring to a different frame in the argument ….’ – For about one hour, the discussion goes on, about the meaning of different code sentences, what should be re-coded and which online forums could already have some reliable software package available. More code is typed and deleted, found online and copied and rewritten.

Bewildered, I look to the right, where another three engineers, Giulia, Fabian and Leonie, sit at a desk in front of a Baxter robot, the size of about a human, frosted red surface and a rectangular monitor as its head, three monitors connected to the same keyboard. Their glance is seemingly focused, and Giulia appears to vigorously create text on an otherwise dark screen.

Our account follows two engineers as they were writing software to make the robot learn the position of a camera and the object it holds, using existing software packages and codes they could retrieve from online webpages and other sources.

At first, such activities seem unsurprising. After all, improving technology on the basis of existing technical knowledge is what we would expect engineers to do. In this activity, though, user ideas were subtly enacted: User images appeared only after design decisions, but they did not feature in design decisions themselves. In our account, we have seen how the engineers made choices as to which online forum to trust, which available code to include, or which marker to use for the video camera. In all of these cases, the engineers’ choices were determined based on existing technical knowledge. For example, a certain marker was considered for which the implementation into the software seemed most feasible, and which would function to recognize an object moved in the robot’s hand. The engineers knew available technical options and requirements for their software, and thus the technological development was influenced by those. Future users were assumed to appreciate the engineers’ decisions, for example that a video camera could identify an object in a robot’s hand by means of an april tag marker.

This activity, which we would call ‘expanding technological possibilities’, was widespread in the laboratories. Throughout our fieldwork, we witnessed engineers coding software or calibrating or improving hardware on the basis of existing technology, for which user images emerged as a sideline. For example, we interviewed two engineers who worked at the robot department on how a robot can better detect and remember different objects in the environment. We asked them why they decided to focus on improving the ability of robots to detect such objects, and, subsequently, who could be users for this. These are their replies:

Alexandru: ‘You find lots of labs working with these robots. But really there are only very few that are working on robots that can operate for extended periods of time. So our robot is supposed to work for four months … and then that leads to lots of interesting questions like … can the robot start with no information and figure out that “OK all of this is static, all of this is dynamic and I saw this person here today and maybe he’s going to be there tomorrow.”’

…

Erik: ‘Imagine, you have a robot at home, aiding the person with dementia. For example, … the person might have a problem remembering the location of certain objects. And then I can imagine that it will definitely be useful with such a thing.’

(excerpt from interviews - robot department)

Ideas about users were created once decisions about technology improvements have already been made. Prior to the advances by the two engineers, the technology allowed only for a short-term deployment of the robot in a given setting. With this technology, the engineers could not actually deploy the robot to assist people with dementia in the elderly care home. The improved technology then allowed the robot to remember objects over ‘extended periods of time’. Only after the improvement was made was it possible for the engineers to conceive of older people with dementia as potential users of their robot.

Likewise, in the speech department, our observations also show how user images emerged after novel design decisions. Here, the engineers worked with an existing commercial robot head and sought to further improve its trustworthiness:

Ida: ‘How can we make … [the robot head] more trustworthy?’

Matthew: ‘We … thought of maybe giving it the look and voice of an older person…’ Everyone in the meeting room nodded.

…

Fredrik: ‘Yes, it is kind of new that we now can create different personas, so we should definitely try it!’

(excerpt from observed conversation – speech department)

The engineers connected the issue of trustworthiness with the possibility of exploring ‘new’ technological advancements of visually adjusting the robot’s appearance to different ‘personas’. In this process, they then articulated the idea that they could enhance the robot’s trustworthiness by giving it the look of an ‘older person’. Again, ideas about the user were enacted together with technological development activities: As the engineers worked to improve robot trustworthiness based on new possibilities to alter personas, they began to enact implied representations of users who appreciate such personas.

In the examples above, it becomes clear how the activity of expanding technological possibilities co-constituted user representations: As our excerpts illustrate, user images were drawn upon because they could easily map onto what a new technology might provide. Implicitly, then, users were seen as *receivers* of new technologies, to be selected or chosen, and who would not have to play a role in the engineers’ initial technological development decisions. At the same time, expanding technological possibilities assumed users who would appreciate these newly developed technological features – regardless of whether this group included users of a video camera identifying an object, older people with dementia, or users who would require increased trustworthiness.

In sum, in both departments, we witnessed how the engineers often worked to augment the abilities of existing robot software and technology. Our observations show how this activity of expanding technological possibilities concomitantly constituted images of users who could make sense of what these new technologies might offer. In our cases, these representations referred to images of users who would gladly receive and appreciate new technological features envisioned by the engineers.

### Universalizing applicability

Four engineers gather around a monitor. On it, a webpage is displayed, black font on a light grey background, fragmented and preliminary, structured into different categories and segments. ‘Robot internal wiki’ is written across the upper edge of the screen; underneath, a caption reads: ‘The purpose of this wiki is to stimulate as much as possible code reuse and the exchange of ideas’. ‘Good job’, Fabian says to Patricio and Giulia, and scrolls down the page. On a sidebar menu, headlines are labelled, ‘tutorials’, ‘frequently used resources’, and ‘repositories’. While cursorily scanning the page, Tiago comments: ‘I wonder. If you are a complete newcomer, is it really understandable for them? … I suggest that … we change the semantics and explain a bit better ….’ – ‘We could use tags to indicate if a code is universally transferable to other robots’ – ‘Mhm, and for the codes, also template descriptions that explain what to accomplish with each code…’ – ‘Yeah, readme-files!’ The gathering dissolves as the engineers spread to different parts of the laboratory. …

I follow Tiago and Patricio, who suggested creating a template description and, at some distance, sit down behind them. Tiago begins to handle keyboard and mouse, while Patricio is seated besides and gazes at the screens, a PR2 robot behind them. Various windows open on the screens, stacked one row behind the other, flashing codes and displaying texts and projecting graphs and images and videos. ‘I make this … so that it can be used simply by everyone…’ – ‘… a universal code that works with all robots and their different parts …’ Tiago opens a text file, and starts typing: ‘This tutorial will guide you through the process of setting up a monocular usb camera in a way that is ROS compatible.’ Gradually, the text file fills, as the engineers browse through the windows, skim through forums, copy and paste hyperlinks and codes, take screenshots of video feeds, concoct, discuss and write, about potential errors, the camera’s rectified image, node names and different packages that can be used as they are suitable for all robots. …

On another day, Rodrigo moves a computer mouse to open the internal webpage, and, under the section ‘tutorials’, finds a caption: ‘How to use and calibrate cameras using ROS’. He clicks on it and follows the steps for his Yumi robot.

Thus Tiago and Patricio created a software tutorial applicable to a broad variety of different robots and use scenarios, and the software was seen to be used by another engineer in another situation. This activity of ‘universalizing applicability’ was common in the robot department. We made several observations of engineers seeking to ensure that their developed technologies would reach a broader audience, for example by spending a great deal of time setting up a webpage, or by creating tutorials and readme files. And we often saw engineers writing code and robot applications as broadly as possible so that it could be used in many different circumstances and by many different people.

Our observations also indicate how the activity of universalizing applicability was connected with particular ways of thinking about the users: The image was that users would have pretty much the same needs and would not require specific attention for adjustments, and that plenty of users with similar requirements would exist. It was not infrequent, hence, that multiple users were imagined for one and the same technology, without that adaptations would be necessary. For example, one time, an engineer talked about the use possibilities of an algorithm he had developed:

Fabian: ‘It can be used in many different situations. In this case, specifically, I wrote an algorithm that can learn over time. So, they have an electricity fuse in a company, and they would like to use a Kuka robot to work with this electricity. And in a big company, what the robot needs to do with the electricity buttons needs to change over time. So they have to learn new behaviour. … [A]nother example could be, for example, the opening of a glass. In waste treatment …. My software could help the robot learn how to open different glasses differently.’

(excerpt from observed conversation - robot department)

Fabian perceived ‘many different situations’ in which his technology could be employed: apart from the actual user, the electricity company, and another user in the waste treatment sector. While universalizing applicability, the possibility of differences between electricity companies and waste treatment enterprises faded into the background. Instead, the already-developed software worked well as a blueprint that could be employed in very different use scenarios. Hence, those two use cases were imagined to display similar needs, so that certain alterations of the software were not necessary.

In sum, in the robot department, the engineers were busy creating technologies that could be used by different groups of users. This activity of universalizing applicability collaterally involved a set of user representations. These images were sketchy and tentative, but they assumed, implicitly, that users from a variety of different context would share certain traits or needs. In contrast, the speech department dealt with one specific use scenario, so no traces of this activity have been observed there.

### Making robots human-like

Classical music plays from a smartphone in Fabian’s hand. Four other engineers around him stare at the smartphone’s screen streaming a youtube video. In the video, two men sit next to each other and, facing the camera, bump each other’s fists in various alterations. At the bump labelled ‘explosion’, Fabian pauses the video. ‘Like this!’ Fabian moves his hand in gestural mimicry of the bump on the screen, as he clenches his fingers into a fist, abruptly releases and frantically sways them through the air as though his hand was actually exploding. A peal of laughter pervades the air.…

Tiago and Fabian fetch a mechanical hand, five fingers and a wrist, phalanges of black plastic and a gleaming, metallic blue skeleton. The hand has all the joints a human hand also has, fixed with numerous silver screws, and wired to a circuit board at its bottom. ‘Let’s remove the parallel gripper first …’, Patricio says. Giulia and Leonie drift towards the Baxter robot and begin to fiddle around something that juts out of the robot’s arm, two-fingered, robust plastic. Cable by cable, screw by screw, and with the squeamish precision of a surgeon, they unplug the gripper and, in an equally scrupulous fashion, mount the mechanic hand onto the arm. ‘That will be difficult, making the robot hand move like in the video …’

The engineers gather at the computer monitor facing the robot, and Giulia opens a virtual window mapping all of the robot hand’s fingers and joints. Leonie stands up, flexes the robot hand’s fingers into a fist, grabs the robot’s arm and purposefully thrusts it forward. Simultaneously, Giulia observes the monitor running down a code, and, towards the end of the movement, hurriedly adds some more code. She presses enter and, as if by magic, the robot arm glides forward without any guidance. ‘Not yet natural enough …’ Leonie repeats, doggedly curving and shoving, adjusting and pushing, fingers and arm of the robot. On and on, Leonie swings and steers, Giulia reprograms and executes, and the robot budges arm and hand in imitation until, eventually, the engineers agree that pace and bump are satisfactory enough.

Our account covers an episode in which the engineers replaced a two-fingered gripper with a five-fingered robot hand, which in many functions resembled a human hand, and subsequently taught the robot to move its arm hand so that it can fist-bump like humans do. Various times, we have observed such instances of how engineers developed robots to equal a human as closely as possible, and inscribed human-likeness into the robot. In another example, an engineer developed a software code so that its arms and hands would include human-like muscles and move like a human, as our observer joined him:

I sat down and asked: ‘What is this code for?’

Tiago: ‘Reorientation … so it allows a robot to lift a pen and twist it in the hand.’He showed me the gesture on his own hand, shifting the pen slowly over the even surface of the table and then twisting it.

Me: ‘How does the code work?’

Tiago: ‘How it works is … Initially, the robot knows only the position of his own arms and hands, but it doesn’t know the length of the pen. However, there are some sensors that can measure the force, angle and velocity … With these, the software can calculate the length of the pen…’

Me: ‘I see. So that I understand that correctly … what can an engineer do with the software then?’

Tiago: ‘There are algorithms for each feature of the arm of the robot … I already programmed these algorithms … they work like human muscles …. [F]or each feature of the arm, they determine how it should react during the process of turning around a pen. And with this software, you only have to put in the velocity, force and angle and then the software does the rest.’

(excerpt from observed conversation - robot department)

In other instances, we made observations of how engineers repeatedly worked to make the robot lift the orange glass like a human, take a mobile phone like a human, and arrange things into containers like a human. Very often, actions were directed towards creating an ever more human-like robot. This term was partially articulated by the engineers themselves, as they frequently reinforced the overall goal of a ‘human-like’ robot. This activity was also present in the speech department:

Matthew: ‘That’s also something that I’ve been thinking of… because we also want [the

robot head] … can be an emotive human companion… How can we measure feelings?

How can [it] … understand human feelings and respond naturally?’

Fredrik: ‘There are some sensors that can measure the heart-rate … And with that, we

can infer some feelings ….’

Oscar: ‘One time, we set up this video camera to measure the sweat and stress

patterns in the human face. That actually worked quite well ….’

(excerpt from observed conversation – speech department)

Having witnessed how the engineers often acted in ways that would make their robots more like a human, we began to wonder about the users of such technologies. Asked about the users of a robot with the ability to lift elements like a human, an engineer elaborated:

Fabian: ‘For example, to replace factory workers one day, or help on Mars to lift some stones or other materials.’

(excerpt from observed conversation – robot department)

This brief passage epitomizes how the activity of creating human-like robots had implications for how the engineers imagined users. Their image was that once a robot is like a human, it can then *replace* humans. According to the engineers, replacing humans with robots could help to reduce inefficiency, imprecision, the burden of heavy or, as Patricio claimed in a different conversation, ‘boring’ labor. This would then be appreciated by users such as factories or space agencies. In other instances, we heard how the engineers implicitly assumed that robots could replace parts of the work of social care workers in elderly care homes:

Rodrigo laughed: ‘It would be an awesome research paper about using a robot to unroll toilet paper.’

The engineers laughed.

Giulia remarked: ‘But there would be no user for this!’

Rodrigo replied: ‘Well, elderly care …’

(excerpt from observed conversation – robot department)

Clearly, while primarily designing robots to become more like a human, the engineers also inscribed in their images various contexts of use in which the robot would replace human work, in this case in the context of social care work. The notion that robots could replace humans, or at least parts of a human’s task, was also prevalent in the speech department. In one of the observed meetings, the engineers discussed how their robot head can be better social support:

Ida: ‘The goal of this project is to use both explicit memory tests … as well as implicit inferences from nonverbal and verbal signals … to determine whether the patient has Alzheimer’s. … But we also want that this agent can be of social support …’

Matthew: ‘Yes, we were thinking to give the robot the look of a doctor to create authority …’

(excerpt from observed conversation – speech department)

Here, the engineers’ ambition was that the robot head could assume the task of a human doctor in diagnosing Alzheimer’s, and it could incarnate a human doctor’s authoritative presence. Their idea was that once the robot face looks like the face of a ‘doctor’, then it could actually radiate the same ‘authority’ as a human doctor. Implicitly, would appreciate having a robot doctor at their home that could diagnose Alzheimer’s rather than a human doctor.

In sum, in both departments, the engineers were making robots more human-like. This activity entangled images of users who would, for at least some tasks, appreciate robots rather than real-human doctors, factory workers, care helpers or nurses.

## Discussion: Re-conceptualizing the constitution of user representations

### Image-evoking activities and user image landscape

Engineers, whether or not they are explicitly concerned with users, create images of users as a byproduct of routine and everyday activities. We have presented four such activities specific to our two cases: making robots human-like, expanding technological possibilities, proficiency demarcation, and universalizing applicability. In our view, each of these activities evoked a number of user images. For example, when making robots human-like, the engineers tried to endow their robots with human capacities, such as the ability to move like a human, look like a human, and respond to human emotions. The engineers mobilized images of users that would appreciate such a human-like robot, and these user images were grounded in the very activity the engineers were involved in: They articulated that users would appreciate a robot replacing humans, or parts of human work, precisely because they have been working on and configuring robots to resemble humans. In other words, the activity ‘making robots human-like’ evoked images of users appreciating robots to replace humans or human work. Similarly, in the activity ‘universalizing applicability’, the engineers sought to build technologies and different software algorithms in such a way that they would speak to many different user groups. In doing so, they assumed images of users who would have similar needs and requirements, so that special attention or adjustments would not be necessary. Such imagined users were, for instance, different businesses from the electricity and waste treatment sector. Again, these images emerged out of the activity the engineers were engaged in: The engineers envisioned users who would possibly have many needs in common, exactly because they were working to broaden the field of applicability for their technologies.

Next to this, in the activity ‘expanding technological possibilities’, the engineers were pushing technological boundaries, for instance, to develop the robot’s capacity to remember the location of objects over an extended period of time. They then mobilized the image that older users with dementia would appreciate a robot that would help them remember the location of different objects. Once again, these results show how user images were enacted together with the activity in which the engineers were involved: The idea that people with dementia would be open to receive and appreciate a robot to remember objects related very well to the technical opportunity on which they were working. Hence, the activity ‘expanding technological possibilities’ evoked a number of images of users as receivers and appreciators of what the engineers regarded as possible technological improvements. Finally, in the activity ‘proficiency demarcation’, the engineers drew on images of users as outsiders to the technical world, without particular technological competency, and who could be more reliably understood by specialists. Again, those images were intertwined with the very activity in which the engineers were involved. So, the activity ‘proficiency demarcation’ also evoked an array of images of users who are situated beyond the technical sphere, and that can be represented legitimately by user experts.

We propose to call such activities *image-evoking*: An image-evoking activity is one in which the engineers are collectively engaged and that enacts a number of user images related to this activity, without explicitly aiming for the creation of user images. Image-evoking activities exhibit three typical features: First, image-evoking activities offer certain user images to engineers while downplaying the relevance of others. For instance, ‘making robots human-like’ implicitly caused engineers to think of users who do not like machine-like robots and who would appreciate robots that replace humans. Without the prefiguration of the robot as human-like, the user image of the robot replacing a human in social interactions would have been less tangible. Second, each image-evoking activity offers multiple contingent user images. By this, we mean that image-evoking activities make certain user images possible, but do not prescribe them. Hence, in our case, the engineers could link a human-like robot to a diverse range of use scenarios, including elderly care, factory work and missions to Mars. Image-evoking activities create user images at the intersection of other contextual factors, such as cultural features, joint decisions, selection of literature and goals set by superiors or funding agencies. Previous work has highlighted that the broader organizational context in which designers operate may configure their actions (Hyysalo, 2006; Mackay et al., 2000). We add to this a perspective on activities as a key process through which this context matters in conjuction with user images. In our conceptualization, the distinction between context and the actions of designers breaks down to make way for activities as the main focus for understanding user images. Third, image-evoking activities are implicit practices. That is, the specific outcomes of image-evoking activities can go unnoticed and be taken for granted. During our fieldwork, none of the engineers doubted the specific user images that they articulated, and they did not deliberately question the origins of the images. Making image-evoking practices visible as such seems to be a good starting point in order to make engineers more reflective about how their work is relevant in framing users and future scenarios for use (Rip and Schot, 2002).

The concept of ‘image-evoking activity’ invites us to look further than identifying and categorizing often ideal-typical user representation techniques (Akrich, 1995) or sources of user images (Hyysalo, 2009; Peine and Herrmann, 2012). In image-evoking activities, user images can be *collaterally* evoked during everyday design activities, so that it is difficult to disentangle sources from activities. Rather, image-evoking activities emphasize how sources and techniques themselves can be constituted in design activities. As such, the concept of ‘image-evoking activity’ draws attention to the contingent and implicit ways user images are enacted as part of ongoing practices within a complex socio-material network. Empirically investigating image-evoking activities, therefore, might help researchers and practitioners to grasp how and why particular user images (and not others) exist in design, whilst also attending to the difficulties associated with introducing new activities and user images into the laboratories.

We also argue that, in our empirical accounts of the messy reality of engineering design work, a neat separation of different image-evoking activities was not apparent. Rather, image-evoking activities seemed to operate jointly, and hence should be studied in conjunction. For the engineers, all image-evoking activities work together to constitute a *user image landscape:* a large but restricted realm of user images that are perceptible to the engineers based on activities in which they are engaged. Like a natural landscape, the user image landscape depicts a certain terrain of user images that are within sight. Depending on the viewpoint, there might be some difference in depth, with some images being more perceptible than others, and there might be some images that are entirely out of sight. As such, a user image landscape is woven together out of the fabric that is created by the activities the designers are partaking in. Its constitutents resonate with the tools and machines engineers and designers are working with, existing prototypes and technologies, their vocabulary and grammar, their biographies, circulating stories, dreams and ambitions.

Bucciarelli’s (1988, 1994) concept of ‘object worlds’ is related to the perspective we are trying to develop here. Bucciarelli argues that the engineers operate in different worlds with different objects, which he called object worlds – ‘worlds of technical specializations, with their own dialects, systems of symbols, metaphors and models, instruments and craft sensitivities’ (Bucciarelli, 1988: 162). An object world encompasses a certain language, a way of understanding and thinking about a certain object. For example, in the object world of a mechanical engineer, a robot is stable, while in the world of computer engineers, it is configurable. Like the ‘user image landscape’, the ‘object world’ refers to the practices of engineers and how they are inherently connected to objects and technical expertise. Bucciarelli’s term highlights how engineers enact *objects*. The concept of the ‘user image landscape’ highlights how engineers, together with objects, enact *users and types of use* as well. Engineering practices can be constitutive of object images and user images.

User image landscapes may also differ from lab to lab, depending on different activities. For example, in contrast to the engineers in the robot department, the engineers in the speech department did not engage in the activity ‘universalizing applicability’. This difference manifests itself in the engineers’ user image landscape. In the speech department, the engineers’ user image landscape contained images in relation to the human-like ideal of the engineers (making robots human-like), images in relation to what the engineers deemed possible based on the existing technology (expanding technological possibilities), and images in relation to the engineers’ distinction between non-technical and technical proficiencies (proficiency demarcation). In the robot department, the engineers’ user image landscape *also* comprised images of users with similar needs regarding the same technology (universalizing applicability).

It follows that user image landscapes are specific to labs and locales. What if the engineers in the robot department were working with a robot that looked like a seal, a dog, or simply a machine instead of like a human? Then, the engineers might be engaged in different image-evoking activities that would constitute a different user image landscape. Furthermore, what if the engineers in the speech department were more engaged with psychology literature? How would they have thought of an improvement in trustworthiness? Would they still maintain the image that an ‘older face’ would increase the trustworthiness of older users into their robot head? If the engineers at the speech department were more engaged in image-evoking activities related to psychology, they probably would have formed other user images. Location-specificity thus forms a central feature of the user image landscape.

### Implications: Design settings, social impact and future research

Our ethnographic analysis has demonstrated the emergence of user images as both constituted by, and intricately linked to, everyday activities of engineers. We have proposed two concepts to help researchers understand the co-constitution of engineering work and user images: image-evoking activities and user image landscape. Our analysis has implications for utilizing user involvement (Hartwick and Barki, 1994; Ives and Olson, 1984; Kujala, 2003), participatory (Schuler and Namioka, 1993) or ethnographically informed design (Anderson, 1994) to capture more knowledge about the user: Our results suggest that such interventions would need to be reconciled with the practical realities in the laboratories; they would have to be built within a complex world of design activities, technology and code. In this regard, we believe that understanding how user images are co-constituted with design practices is a good starting point from which to provide recommendations that are more sensitive to the complexities of engineering work.

It has frequently been argued that the engineers’ preoccupation with ‘technical’ issues may cause them to exclude more ‘social’ aspects of their work (Faulkner, 2000; Hacker, 1989). We do not wish to condemn the engineers’ outlooks (Stewart and Williams, 2005). They are involved in complex design activities, and these limit the opportunities to develop ideas about their technology’s social impact. At the same time, our findings do show that, while developing new technology was the primary concern of the engineers, the engineers implicitly developed images of possible users for their technology as the work progressed. We found that during many situations in the field, user images emerged in the aftermath of established goals or existing objects. As such, our results reinforce Šabanović’s (2010) warnings that the current perspective of robot scientists on the relationship between robots and users in society is based on a view of technological determinism. Users were imagined through the mold prepared for them by the emerging technologies, and their ability and willingness to fit this mold was hardly doubted.

The implicit imagery of users in our robot cases included surprisingly specific statements about socio-material futures. In particular, we witnessed how actions related to making robots more human-like gave rise to use scenarios in which robots would replace humans. Such specific articulations of future arrangements with technology can be made obdurate and mobile through the robots that are being built (while alternative arrangements may never materialize in other robots). Our study offers some evidence that shows how such articulations may materialize in the design of robot prototypes. Of course, we do not know yet to what extent robots eventually will replace humans. But the ways such scenarios are imagined in the laboratories and inscribed into technologies at least does not preclude the possibility of irrational, negative outcomes (Ritzer, 1983). Dehumanization of social relations and replacement of human work through robots may silently become a self-fulfilling prophecy.

Finally, we have developed a perspective on user representations that moves away from viewing them as the combination of some ideal-typical sources toward thinking about them as enabled and constrained through design practice. During our ethnographic analysis we found that questions arose that were not apparent prior to this analysis. Would image-evoking activities and the user image landscape evolve across multiple instances of design phases? Would those change or adapt when having contact with users? And how would this affect the design results? Based on our findings, we could hypothesize that transformations are possible if activities change. But this leaves open how such change, adaption or transformation may take place in practice, what would set such changes in motion, and to what extent this would influence the design outcomes. If one is to learn more about the constitution of scripts and user representations, answering these questions appears to be a fruitful path.
